# Predictors of Emergency Interventions in Acute Airway Obstructive Diseases: A Retrospective Single-Center Observational Study

**DOI:** 10.7759/cureus.71031

**Published:** 2024-10-07

**Authors:** Naoya Suzuki, Tomoki Doi, Takeru Abe, Takahiro Michishita, Masayasu Gakumazawa, Ichiro Takeuchi

**Affiliations:** 1 Department of Emergency Medicine, Yokosuka Kyosai Hospital, Yokosuka, JPN; 2 Advanced Critical Care and Emergency Center, Yokohama City University Medical Center, Yokohama, JPN

**Keywords:** acute airway obstruction, airway management, critical emergency medicine, early warning score, physical findings

## Abstract

Background: The factors related to emergency intervention for internal medicine conditions leading to airway obstruction are not clear.

Objective: We aimed to identify factors associated with emergency interventions in acute airway obstructive diseases (AAODs).

Methods: This is a retrospective observational single-center study. We defined AAODs as acute epiglottitis, peritonsillar abscess, tonsillitis, pharyngitis, oral floor abscess, neck abscess, angioedema, Lemierre's syndrome, hemoptysis, and airway foreign body. We compared the group required airway interventions (intubation, cricothyroidotomy, tracheostomy) with the group treated conservatively admitted to Yokosuka Kyosai Hospital, Japan (tertiary referral hospital) for AAOD between April 2012 and March 2022.

Results: Two hundred fifty-five patients were admitted for AAOD, 104 patients were excluded, and 150 patients (39 intervention group, 111 conservative group) were analyzed. Univariate analysis revealed significant age differences (74(61-78) vs 67(31-76), p<0.01), Glasgow Coma Scale (15(14-15) vs 15(15-15), p<0.01), respiratory rate (24(20-30) vs 20(16-22), p<0.01), National Early Warning Score (NEWS) (6(3-9) vs 3(1-5), p<0.01), Sequential Organ Failure Assessment (SOFA) score (2(1-4) vs 0(0-2), p<0.01), stridor (26% vs 2%, p<0.01), dysphagia (41% vs 21%, p=0.02), drooling (18% vs 3%, p<0.01), frequent suctions (6% vs 0%, p<0.01), airway examination abnormalities (AEAs) (69% vs 32%, p<0.01) and diagnosis (p<0.01). Multivariate logistic regression analysis indicated AEA (OR=9.41, 95%CI 3.66-24.2), upper airway diseases (OR=5.74, 95%CI 2.12-15.6), and SOFA score (OR=2.88, 95%CI 1.06-7.83) were predictors for intervention. However, the sensitivity and specificity of AEA were 0.69 (95%CI 0.52-0.83) and 0.69 (95%CI 0.59-0.77), respectively.

Conclusions: AEAs were associated with a high risk of airway interventions in AAOD. Nevertheless, the sensitivity and specificity were insufficient.

## Introduction

Airway obstruction constitutes a life-threatening emergency and presents significant challenges for clinicians, as managing this condition demands impeccable precision in decision-making [1]. Many patients with airway obstruction require interventions outside the operating room, each presenting unique risks [2]. Additionally, emergency interventions necessitate differential diagnoses, considering each patient's distinct pathology. Clinicians must evaluate numerous factors, including age, comorbidities, level of consciousness, ability to lie supine, and the degree of obstruction [1].

The ABCD approach (Airway, Breathing, Circulation, Dysfunction of the Central Nervous System) is widely recognized in trauma care but is not as prevalent in internal medicine [3]. A variety of conditions can precipitate airway emergencies, with endogenous diseases such as croup, supraglottitis, epiglottitis, neck abscess, Ludwig's angina, angioedema, tumors, and foreign bodies being significant contributors [1]. Additionally, peritonsillar abscesses, tonsillitis, pharyngitis, and hemoptysis may also lead to airway obstruction [4,5].

Cases necessitating airway interventions are rare and challenging to compile. Some studies suggest that acute epiglottitis and Ludwig's angina are indications for tracheal intubation [4,5]. However, there are no comprehensive reports on urgent airway interventions, including tracheal intubation, cricothyroidotomy, and tracheostomy. This study aims to investigate the diseases that cause airway obstruction and the factors predicting the need for airway interventions.

## Materials and methods

Study design and participants

Acute airway obstructive diseases (AAODs) were defined as acute epiglottitis, peritonsillar abscess, tonsillitis, pharyngitis, Lemierre's syndrome, neck abscess, angioedema, hemoptysis, and airway foreign body. We included patients admitted to a tertiary referral hospital (Yokosuka Kyosai Hospital, Japan) through the emergency department for AAOD (age >15) between April 2012 and March 2022. Exclusion criteria encompassed do-not-attempt-resuscitation (DNAR) orders, do-not-intubate orders, out-of-hospital cardiac arrest, coma (Glasgow Coma Scale (GCS) <9), prior intubation, tracheostomy, duplication, and data insufficiency.

Ethical statement

This single-center retrospective observational study was approved by the Yokosuka Kyosai Hospital Institutional Review Board (number 22-55). Informed consent was waived because of the retrospective and observational nature of this study.

Outcomes

The primary objective of this retrospective study was to identify predictors of emergency airway interventions. The intervention group (Group I) was defined by the presence of one or more of the following: tracheal intubation, cricothyroidotomy, or tracheostomy. The conservative group (Group C) was defined by the absence of interventions. Predictive variables included age, sex, body mass index, Charlson comorbidity index, GCS, respiratory rate, oxygen saturation, heart rate, blood pressure, body temperature, National Early Warning Score (NEWS), Sequential Organ Failure Assessment (SOFA) score, and physical examination findings (stridor, hoarse voice, dysphagia, drooling, and frequent suctioning (at least once every 10 minutes)). Airway examination abnormalities (AEAs) were defined as the presence of one or more of the following: stridor, hoarse voice, dysphagia, drooling, and frequent suctioning. Additional variables included time of hospital visit, transport method, and diagnosis. Diagnoses were categorized into two groups: lower airway diseases (hemoptysis) and upper airway diseases (all others). Furthermore, we compared emergency care unit (ECU; equivalent to a high-dependency care unit), intensive care unit (ICU) admission, length of stay in ECU/ICU, hospital length of stay, ventilator days, in-hospital mortality, cerebral performance category at discharge, unexpected cardiac arrest, and unplanned ECU/ICU admissions between Group I and Group C.

Statistical analysis

Continuous variables are presented as median with interquartile range (IQR) or mean with standard deviations. Categorical variables are expressed as numbers and percentages. For comparing the two groups, the Mann-Whitney U test was employed for continuous variables, while Pearson’s chi-square test or Fisher's exact test was utilized for categorical variables, as appropriate. Univariate and multivariate logistic regression models were applied to evaluate the association between Group I and Group C.

All statistical analyses were performed with EZR (version 1.61; Saitama Medical Center, Jichi Medical University, Saitama, Japan), which is a graphical user interface for R (The R Foundation for Statistical Computing, Vienna, Austria). More precisely, it is a modified version of R commander designed to add statistical functions frequently used in biostatistics [6]. A two-sided p-value <0.05 was considered statistically significant.

## Results

**Figure 1:**
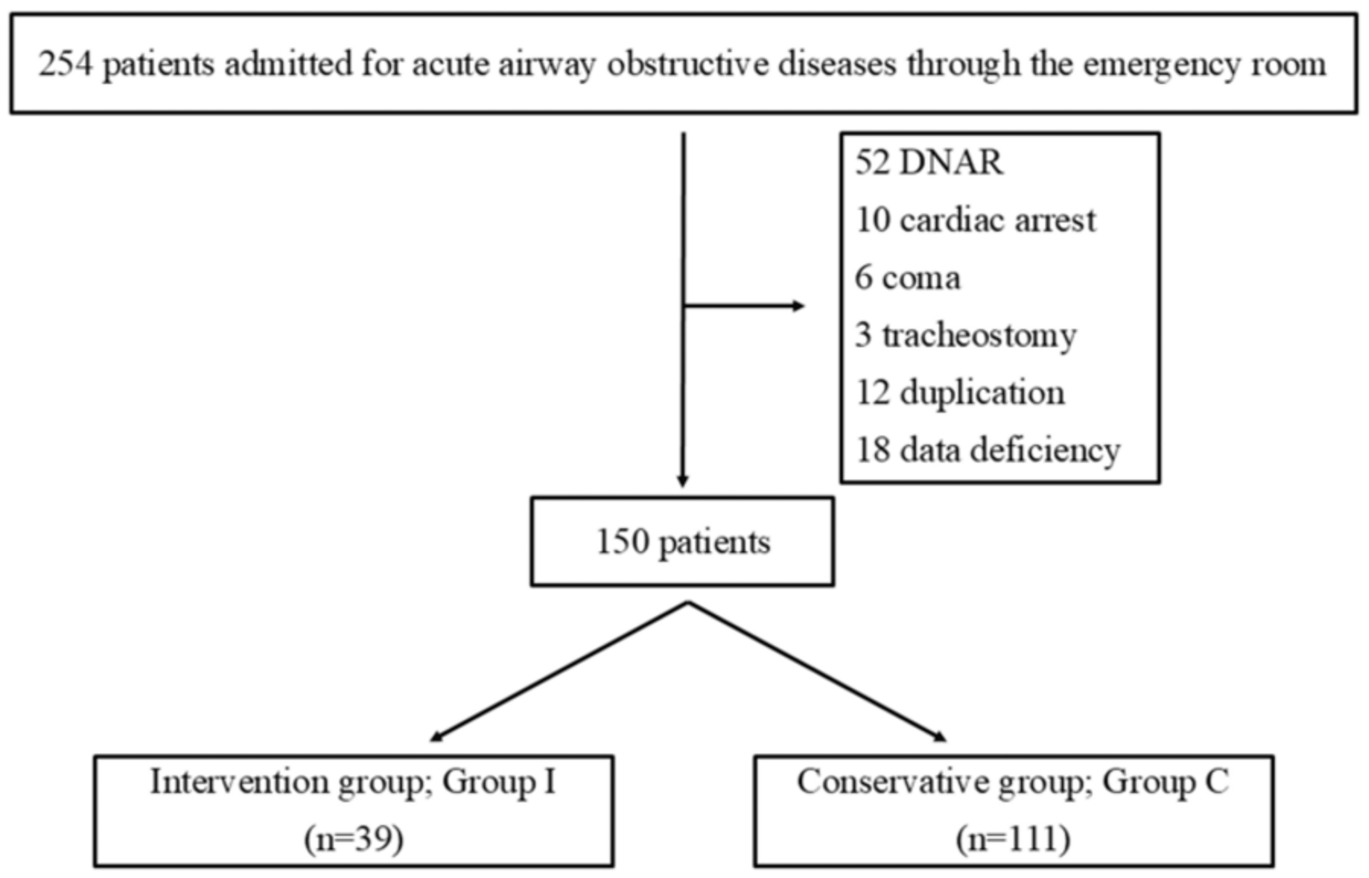
Patient flowchart DNAR: do not attempt resuscitation

Between April 2012 and March 2022, 133,691 patients visited our emergency department and 254 (0.002%) were hospitalized for AAOD. One hundred four patients were excluded (52 did not attempt resuscitation, 10 were out of hospital cardiac arrest, six were in coma, three tracheostomy, 12 duplications, and 18 data deficiencies), and 150 patients were analyzed. Group I had 39 patients (26%) and Group C had 111 patients (74%) (Figure 1).

Baseline characteristics are shown in Table 1. In Group I, 36 (93%) were intubated, two (5%) had cricothyrotomies, and 16 (41%) had tracheostomies. The significant differences between the two groups in the univariate analysis were age (74(61-78) vs 67(31-76), p<0.01), GCS (15(14-15) vs 15(15-15), p<0.01), respiratory rate (24(20-30) vs 20(16-22), p<0.01), NEWS (6(3-9) vs 3(1-5), p<0.01), SOFA score (2(1-4) vs 0(0-2), p<0.01), stridor (26% vs 2%, p<0.01), dysphagia (41% vs 21%, p=0.02), drooling (18% vs 3%, p<0.01), frequent suction (6% vs 0%, p<0.01), AEA (69% vs 32%, p<0.01). Group I had significantly more upper airway diseases and Group C had more lower airway diseases (p<0.02).

**Table 1:** Patient characteristics IQR: interquartile range, NEWS: National Early Warning Score, SOFA: Sequential Organ Failure Assessment, NA: not applicable, ICU: Intensive Care Unit, ECU: Emergency Care Unit *BMI data were missing for one patient in the Intervention group and nine patients in the Conservative group

Characteristics	All patients (n=150)	Intervention group (n=39)	Conservative group (n=111)	P-value
Age, y, median (IQR)	70 (40-77)	74 (61-78)	67 (31-76)	<0.01
Males, n (%)	89 (59)	25 (64)	64 (58)	0.61
Body mass index (kg/m^2^), median (IQR)*	23 (20-25)	23 (19-25)	22 (20-26)	0.66
Charlson comorbidity index, median (IQR)	1 (0-2)	1 (0-2)	1 (0-2)	0.07
Glasgow coma scale, median (IQR)	15 (15-15)	15 (14-15)	15 (15-15)	<0.01
Respiratory rate, median (IQR)	20 (17-24)	24 (20-30)	20 (16-22)	<0.01
Saturation percutaneous O_2_, median (IQR)	98 (96-99)	96 (95-98)	98 (98-99)	0.1
Heart rate, median (IQR)	101 (89-113)	105 (90-118)	100 (89-111)	0.29
Systolic blood pressure, median (IQR)	135 (116-160)	138 (119-160)	132 (116-162)	0.99
Diastolic blood pressure, median (IQR)	79 (66-95)	71 (62-96)	79 (70-95)	0.23
Body temperature, median (IQR)	37.1 (36.6-37.9)	37.1 (36.6-38)	37.1 (36.8-37.6)	0.84
Diagnosis, n (%)				0.02
Upper airway diseases	91 (61)	30 (20)	61 (41)	
Acute epiglottitis	8 (5)	5 (3)	3 (2)	
Peritonsillar abscess, tonsillitis	44 (29)	2 (1)	42 (28)	
Pharyngitis	6 (4)	1 (1)	5 (3)	
Neck abscess	16 (11)	14 (9)	2 (1)	
Angioedema	5 (3)	2 (1)	3 (2)	
Airway foreign body	5 (3)	3 (2)	2 (1)	
Others	7 (5)	3 (2)	4 (3)	
Lower airway diseases	59 (39)	9 (23)	50 (45)	
Hemoptysis	59 (39)	9 (23)	50 (45)	
Airway examination abnormalities, n (%)	62 (41)	27 (69)	35 (32)	<0.01
Stridor, n (%)	12 (8)	10 (26)	2 (2)	<0.01
Hoarse voice, n (%)	32 (21)	13 (33)	19 (17)	0.05
Dysphagia, n (%)	39 (26)	16 (41)	23 (21)	0.02
Drooling, n (%)	10 (6)	7 (18)	3 (3)	<0.01
Frequent suction, n (%)	6 (4)	6 (15)	0 (0)	<0.01
NEWS, median (IQR)	4 (1-6)	6 (3-9)	3 (1-5)	<0.01
SOFA score, median (IQR)	1 (0-2)	2 (1-4)	0 (0-2)	<0.01
Airway interventions, n (%)				
Intubation	36 (24)	36 (93)	NA	
Cricothyroidotomy	2 (1)	2 (5)	NA	
Tracheostomy	16 (11)	16 (41)	NA	
Ambulance transport, n (%)	121 (81)	36 (93)	85 (77)	0.05
ICU/ECU admission, n (%)	53 (35)	34 (87)	19 (17)	<0.01
ICU/ECU length of days, median (IQR)	0 (0-3)	8 (5-12)	0 (0-0)	<0.01
Ventilator length of days, median (IQR)	0 (0-0)	5 (2-9)	0 (0-0)	<0.01
Hospital length of days, median (IQR)	8 (5-16)	24 (14-42)	7 (5-11)	<0.01
In-hospital death, n (%)	7 (5)	7 (18)	0 (0)	<0.01
Cerebral performance category at discharge, median (IQR)	1 (1-2)	2 (1-3)	1 (1-2)	<0.01
Unexpected cardiac arrest, n (%)	5 (3)	5 (13)	0 (0)	<0.01
Unplanned ICU/ECU admission, n (%)	3 (2)	3 (8)	0 (0)	<0.01

**Figure 2:**
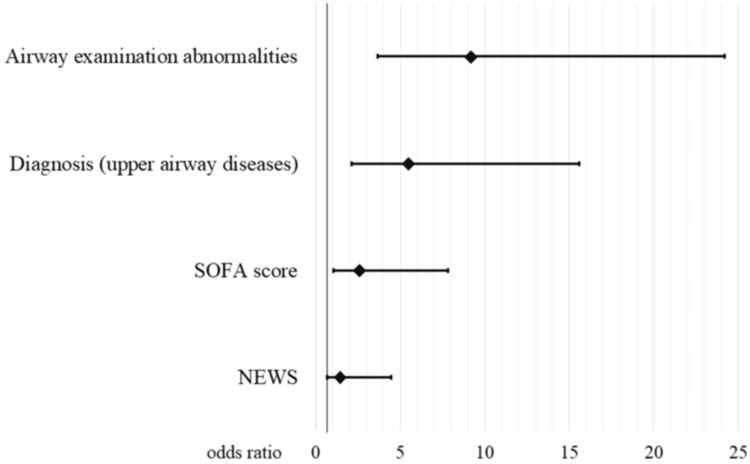
Multivariate logistic regression analysis for airway interventions SOFA: Sequential Organ Failure Assessment, NEWS: National Early Warning Score

Multivariate logistic regression analysis indicated that AEA (odds ratio (OR)=9.41, 95% confidence interval (CI) 3.66-24.2), upper airway diseases (OR=5.74, 95%CI 2.12-15.6) and SOFA score (OR=2.88, 95%CI 1.06-7.83) were predictors for interventions; however, NEWS (OR=1.56, 95%CI 0.66-3.7) were not a predictor (Figure 2).

Nevertheless, the sensitivity of AEA to airway interventions was 0.69 (95%CI 0.52-0.83) and specificity 0.69 (95%CI 0.59-0.77). The accuracy was 0.69 (95%CI 0.61-0.76), the positive and negative predictive values were 0.43 (95%CI 0.31-0.57) and 0.86 (95%CI 0.77-0.93), respectively, and the positive and negative likelihood ratios were 2.20 (95%CI 1.56-3.10) and 0.45 (95%CI 0.28-0.73), respectively. Diagnosis and SOFA scores were also not sufficiently accurate (Table 2).

**Table 2:** Multivariate logistic regression analysis for airway interventions and accuracy OR: odds ratio, CI: confidence interval, PPV: positive predictive value, NPV: negative predictive value, PLR: positive likelihood ratio, NLR: negative likelihood ratio, SOFA: Sequential Organ Failure Assessment, NEWS: National Early Warning Score

	OR (95%CI)	P-value	Sensitivity	Specificity	Accuracy	PPV	NPV	PLR	NLR
Airway examination abnormalities	9.41 (3.66-24.2)	<0.01	0.69 (0.52-0.83)	0.69 (0.59-0.77)	0.69 (0.61-0.76)	0.43 (0.31-0.57)	0.86 (0.77-0.93)	2.20 (1.56-3.10)	0.45 (0.28-0.73)
Diagnosis (upper airway diseases)	5.74 (2.12-15.6)	<0.01	0.77 (0.61-0.89)	0.45 (0.36-0.55)	0.53 (0.45-0.62)	0.33 (0.24-0.44)	0.85 (0.73-0.93)	1.40 (1.10-1.78)	0.51 (0.28-0.94)
SOFA score	2.88 (1.06-7.83)	0.037	0.72 (0.55-0.85)	0.47 (0.37-0.57)	0.53 (0.45-0.62)	0.32 (0.23-0.43)	0.83 (0.71-0.91)	1.35 (1.03-1.76)	0.60 (0.35-1.03)
NEWS	1.74 (0.68-4.45)	0.251	0.49 (0.32-0.65)	0.68 (0.57-0.75)	0.62 (0.54-0.70)	0.34 (0.22-0.48)	0.79 (0.69-0.87)	1.46 (0.96-2.22)	0.76 (0.55-1.07)

## Discussion

AEA, upper airway diseases, and SOFA score were predictors for emergency interventions in AAOD, however, NEWS was not a predictor. Furthermore, AEA was the highest risk for airway interventions. Our study found significant differences in age, GCS, respiratory rate, NEWS, SOFA score, stridor, dysphagia, drooling, frequent suction, AEA, and diagnosis. GCS and respiratory rate were considered confounding factors for NEWS and SOFA scores. Physical findings were also confounding factors for AEA. Therefore, we performed a multivariate logistic regression analysis of diagnosis, NEWS, SOFA score, and AEA.

Various predictors have been reported for each disease. In acute epiglottitis, dyspnea and supraglottic extension of the edema were considered risks for interventions [4], and in Ludwig's angina, significant airway swelling, dyspnea, stridor, cyanosis, or worsening airway symptoms were considered risk factors [5]. In angioedema, shortness of breath, drooling, and anterior tongue or pharyngeal swelling were considered risks for interventions [7]. In adults, acute supraglottitis, dyspnea, and stridor were considered risk factors [8]. After all, physical examinations were risk factors for airway interventions and should be considered the most important aspect. It is reasonable to assume that the rate of airway interventions varies by diagnosis. For example, the reported rate of intubation in hemoptysis was 11% [9], which was close to our results (15%). On the other hand, in upper airway diseases, the results were different. In acute epiglottitis, the reported rate was 15% [10], while our result was 62.5%, and in neck abscess, the reported rate was 16.5% [11], our result was 87.5%. This may be related to the small number of our cases and the bias to select the most severely ill patients because they are routed through the emergency department. It is worthwhile to identify predictors of emergency interventions in airway emergent diseases. In the emergency setting, diagnosis is often not made. In addition, some patient’s conditions require interventions before diagnosis can be confirmed by imaging or other modalities. As such, predicting interventions with respect to the overall diseases causing airway obstruction is important in emergency settings. Consequently, we consider that our study to identify risk factors for airway interventions in AAOD, including a variety of diseases, is meaningful.

It is common practice in trauma emergencies to assess the airway, breathing, circulation, and dysfunction of the central nervous system followed by emergency interventions [3]. However, the importance of this approach in internal medicine has not been well recognized. Various scores are used to evaluate the urgency and severity of patient's conditions. The SOFA score is used to predict prognosis in critically ill patients, scoring consciousness, respiration, circulation, renal function, liver function, and blood counts [12]. The PaO_2_/FIO_2_ ratio is used as the respiratory factor, but the airway factor is not specified. NEWS includes the level of consciousness, respiratory rate, SpO_2_, oxygenation, pulse rate, systolic blood pressure, and body temperature as predictors of death and ICU admission [13]. There are no airway factors specified here either. Patients with airway emergencies often present with increased respiratory rate without severe respiratory failure. Therefore, untrained medical providers may not be able to detect abnormalities. Pulse oximetry is a poor indicator of airway obstruction, and a decrease in arterial hemoglobin oxygen saturation reflects decreased oxygen stores in the lungs which is a delayed sign of progressive hypoxemia [14]. Respiratory rate is also often not measured accurately enough [15]. Airway emergencies have rapid courses and must be fully evaluated. However, these scoring methods do not provide a good assessment. Thus, SOFA score and NEWS were not critical predictors in airway interventions.

This study has several limitations. First, this was a retrospective single-center study. Consequently, the number of explanatory variables in the multivariate analysis was limited. It would have been desirable to evaluate not only the presence or absence of AEA but also the number and severity of symptoms. A multicenter study is needed to confirm these results. Second, the exact criterion for tracheal intubation, cricothyrotomy, and tracheostomy had not been determined. This may have led to divergent indications for treatment among physicians. Third, physical findings may have been missed because the check format was not used. However, we reviewed in detail the medical records of non-physicians (nurses, nurse practitioners, physical therapists, speech therapists, and other co-medicals). It may be considered a hypothesis-generating study because of these limitations. In this regard, future prospective studies are needed that can overcome these limitations to provide the highly accurate risk of interventions for AAOD.

## Conclusions

AEA, upper airway diseases, and SOFA scores were associated with airway interventions in AAOD. Although the sensitivity and specificity of AEA were found to be insufficient, the odds ratio for airway interventions was the highest observed in this study. Therefore, conducting thorough physical examinations may prove beneficial.
